# Long-term prognosis of patients with hepatitis B virus–related acute-on-chronic liver failure: a retrospective study

**DOI:** 10.1186/s12876-022-02239-4

**Published:** 2022-04-02

**Authors:** Lu Wang, Wenxiong Xu, Xuejun Li, Dabiao Chen, Yeqiong Zhang, Yuanli Chen, Juan Wang, Qiumin Luo, Chan Xie, Liang Peng

**Affiliations:** 1 Department of Infectious Diseases, Third Affiliated Hospital of Sun Yat-Sen University, 600# Tianhe Road, Guangzhou City, Guangdong Province China; 2 Guangdong Key Laboratory of Liver Disease Research, The Third Affiliated Hospital of Sun Yat-Sen University, Guangzhou, Guangdong China; 3 Key Laboratory of Tropical Disease Control (Sun Yat-Sen University), Ministry of Education, Guangzhou, China

**Keywords:** Acute-on-chronic liver failure, HBV, Aetiology, Prognosis, Risk factors

## Abstract

**Background:**

The long-term prognosis of patients with hepatitis B virus–related acute-on-chronic liver failure (HBV-ACLF) is not well characterised. We assessed long-term outcomes and the associated risk factors of HBV-ACLF patients in southern China.

**Methods:**

We retrospectively analysed clinical data, adverse events, and clinical endpoint events of HBV-ACLF patients treated at our department between January 2014 and December 2018.

**Results:**

A total of 616 (52.3%) patients with cirrhosis and 561 (47.7%) patients without cirrhosis were included. In 973 (83%) patients, the disease was associated only with HBV, while 204 (17%) patients had two or more aetiological factors. The proportion of patients receiving antiviral treatment for HBV was low (20.3%). Further analyses indicated that patients without cirrhosis had a significantly lower 90-day liver transplantation–free mortality and higher 5‐year survival rate than those with cirrhosis (59.5% vs. 27.6%; 62% vs. 36%; *P* < 0.05). Remarkably, self-withdrawal of nucleos(t)ide analog (NA) was an independent risk factor for short-term prognosis. Age, cirrhosis at admission, and platelet level were closely related to long-term prognosis of HBV-ACLF patients.

**Conclusion:**

The proportion of HBV-ACLF patients receiving antiviral treatment is very low in south China. Cirrhosis at admission has a significant effect on both short-term and long-term prognosis. No significant improvement in the short-term prognosis of HBV-ACLF patients was observed compared with previous studies. More comprehensive access to antiviral treatment and long-term surveillance of HBV patients are key imperatives to reduce the incidence of HBV-ACLF and improve the prognosis.

*Trial Registration* The trial was registered at ClinicalTrials.gov (CT.gov identifier: NCT04231565) on May 13, 2020: https://register.clinicaltrials.gov/prs/app/action/SelectProtocol?sid=S0009OZY&selectaction=Edit&uid=U00036P1&ts=2&cx=27seqt

## Introduction

Acute-on-chronic liver failure (ACLF) is a systemic multi-organ dysfunction caused by an acute hepatic insult in the backdrop of previously diagnosed or undiagnosed chronic liver disease or cirrhosis. It is characterised by rapid disease progression and is associated with short-term mortality of up to 40–90% [1, 2]. Hepatitis B virus (HBV) infection is the leading cause of ACLF in China [3]. Previous studies have found that HBV activation and withdrawal of antiviral drugs are the most common causes of HBV-related acute-on-chronic liver failure (HBV-ACLF) [4]. Of note, the incidence of HBV reactivation caused by the withdrawal of nucleos(t)ide analog (NA) has increased in recent years, which is closely related to the short-term prognosis of HBV-ACLF patients [5].

With the popularisation of antiviral drug treatment and artificial liver support system (ALSS), some patients can recover through comprehensive medical treatment. However, some HBV-ACLF patients who have survived the acute injury develop post-necrotic cirrhosis and hepatocellular carcinoma (HCC) after several years [6]. The long‐term prognosis of patients with HBV-ACLF and associated risk factors have not been well characterised. Although there was a study that investigated approximately 200 patients with HBV-ACLF in southern China [7], our study had a larger sample size and focused on the long-term prognosis of HBV-ACLF patients over the past 5 years. Furthermore, we explored the risk factors for disease progression to provide a basis for further diagnosis and treatment.

## Patients and methods

### Study participants

**Fig. 1:**
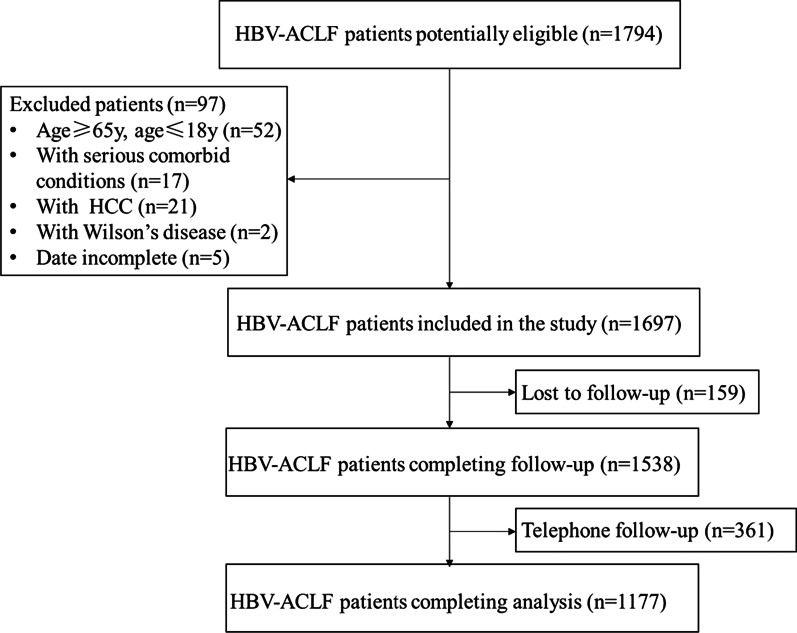
Flowchart of patient screening and selection

This was a retrospective study. We reviewed 1794 patients who had received diagnosis of HBV‐ACLF at the Department of Infectious Diseases, Third Affiliated Hospital of Sun Yat-Sen University, between January 2014 and December 2018. A total of 97 patients were excluded because they did not meet the inclusion criteria. In addition, detailed clinical data of 361 patients were incomplete because of telephone follow-up, and 159 patients were lost to follow-up. Finally, 1177 patients completed follow-up, and their clinical data were analysed in this study (Fig. 1).

The inclusion criteria were as follows: (1) patients with chronic hepatitis B who received diagnosis of ACLF based on the 2014 definition by the Asian Pacific Association for the Study of Liver (APASL); (2) availability of complete inpatient clinical data. The exclusion criteria were as follows: (1) age ≥ 65 years or ≤ 18 years; (2) concomitant presence of liver cancer or other tumours, autoimmune liver disease, or genetic metabolic liver disease; (3) presence of other serious comorbid conditions; (4) lack of timely follow-up.

### Diagnostic criteria

The diagnosis of chronic hepatitis B was based on the guidelines for the prevention and treatment of chronic hepatitis B in China (2015) [8]: (1) HBV surface antigen and HBV DNA positive for more than 6 months; (2) non-invasive examination of liver fibrosis or liver biopsy showing chronic hepatitis.

ACLF was diagnosed based on the definition by the APASL in 2014 [9]: (1) on the basis of chronic liver disease (chronic hepatitis or cirrhosis); (2) coagulation disorders (international normalised ratio (INR) ≥ 1.5 and/or prothrombin activity (PTA) < 40%; (3) severe jaundice (total serum bilirubin (TBIL) ≥ 5 mg/dL); (4) ascites and/or hepatic encephalopathy (HE).

Signs of disease progression for long-term prognosis included clinical adverse events (new decompensated liver cirrhosis, HCC, and ACLF recurrence), liver transplantation, and mortality of patients who survived for more than 90 days.

The diagnosis of liver cirrhosis was based on the following: (1) confirmation by liver biopsy; (2) clinical evidence of decompensation events or varices; (3) supportive imaging evidence, such as liver nodule formation; (4) specific laboratory indicators, such as platelet (PLT) count, albumin (ALB) level, and prothrombin time (PT). If a patient's Child–Turcotte–Pugh (CTP) score was > 7 or there were complications related to portal hypertension, the diagnosis was decompensated cirrhosis [10].

The diagnosis of HCC was based on clinical manifestations and imaging examinations, such as multi-slice spiral computed tomography (CT) and magnetic resonance imaging (MRI) [11].

Liver transplantation (LT) criteria: Model for End-Stage Liver Disease (MELD) score is the primary reference index for evaluating the indication for liver transplantation. A MELD score of 15–40 is the optimal indication for liver transplantation after active comprehensive medical therapy and artificial liver therapy [12].

The definition of serious comorbid conditions: some patients with liver failure have severe extrahepatic chronic diseases, such as diabetes with severe complications, renal failure, and severe coronary heart disease with cardiac function level, which significantly affects their survival.

Self-withdrawal of NA was defined as NA self-discontinuation before the completion of antiviral treatment according to guidelines [8] or against medical advice.

### Follow-up and data collection

In this study, most patients were followed-up with via telephone and during outpatient visits to record disease progression after discharge, including the occurrence of adverse clinical events, death, and liver transplantation. The first patient was enrolled in January 2014 and the last in December 2018. Our follow-up began on May 1, 2014 and ended on May 1, 2020. We retrospectively analysed the following data: age, sex, complications, HBV-DNA, TBIL, ALB, aspartate aminotransferase (AST), alanine aminotransferase (ALT), cholinesterase (CHE), PT, INR, fibrinogen (Fib), alpha-fetoprotein (AFP), white blood cell (WBC) count, PLT count, fasting blood glucose (FBG), blood creatinine (Cr), blood urea nitrogen (BUN), estimated glomerular filtration rate (eGFR), CTP, MELD score, MELD-Na score, and abdominal ultrasound or CT results.

### Statistical analysis

SPSS 25.0 statistical software was used for data processing and analyses. Continuous variables were presented as mean ± standard deviation or median value. Categorical variables were presented as frequency (percentage). The independent-samples *t*-test and Mann–Whitney *U* test were used for the comparisons of continuous variables, and the chi‐square test was used for the comparison of categorical variables. The survival rate was estimated using the Kaplan–Meier method, and between-group differences in survival outcomes were assessed using the log-rank test. Univariate logistic regression analysis and multivariate logistic  regression analysis were used to identify risk factors for 90-day mortality and disease progression. *P* values < 0.05 were considered indicative of statistical significance.

## Results

### Patients’ characteristics

A total of 1177 HBV-ACLF patients were included in the study, including 616 (52.3%) patients with liver cirrhosis (cirrhosis group) and 561 (47.7%) patients with chronic hepatitis (non-cirrhosis group). Patients in the cirrhosis group were older than those in the non-cirrhosis group. Of note, the proportion of patients receiving antiviral treatment for HBV in our cohort was extremely low (20.3%). The ratios of antiviral therapy and self-withdrawal of NA in the cirrhosis group were higher than those in the non-cirrhosis group (*P* < 0.001). The levels of AST, ALT, CHE, Fib, eGFR, FBG, serum Na, PLT, AFP, and HBV-DNA were significantly lower in the cirrhosis group compared with the non-cirrhosis group (*P* < 0.001). Furthermore, compared with the non-cirrhosis group, the cirrhosis group had higher liver severity scores such as the MELD score, MELD-Na, and CTP score (*P* < 0.05) (Table 1).

**Table 1:** Baseline characteristics of the patients

Parameter	Total	Cirrhosis	Non-cirrhosis	*P* value
	(n = 1177)	(n = 616)	(n = 561)	
Age (years)	45.06 ± 10.57	47.38 ± 10.28	42.52 ± 10.30	< 0.001
Sex, male (%)	124 (10.5)	72 (11.7)	52 (9.3)	0.209
Antiviral therapy (%)	239 (20.3)	172 (27.9)	67 (12.0)	< 0.001
NA withdrawal (%)	171 (14.6)	115 (18.7)	56 (10.0)	< 0.001
Drug resistance (%)	21 (1.8)	8 (1.3)	13 (2.3)	0.187
TBIL (µmol/L)	355.57 ± 163.47	359.72 ± 168.67	351.00 ± 157.60	0.361
AST (U/L)	215.50 (107.75, 598.50)	158.50 (92.00, 360.75)	316.00 (136.00, 817.00)	< 0.001
ALT (U/L)	304.50 (88.00, 854.75)	142.00 (65.00, 482.25)	580.00 (177.50, 1316.00)	< 0.001
ALB (g/L)	34.46 ± 17.49	34.65 ± 23.75	34.27 ± 4.80	0.709
PA (mg/L)	38.05 ± 25.42	38.02 ± 24.42	38.07 ± 26.50	0.977
CHE (U/L)	4035.22 ± 1885.37	3697.90 ± 1882.33	4406.34 ± 1819.41	< 0.001
PT (s)	28.03 ± 11.92	28.21 ± 9.10	27.82 ± 14.41	0.572
INR	2.67 ± 2.09	2.68 ± 1.75	2.65 ± 2.41	0.832
Fib (g/L)	1.56 (1.26, 1.96)	1.51 (1.19, 1.83)	1.65 (1.32, 2.09)	< 0.001
BUN (mmol/L)	3.81 (2.74, 5.38)	4.15 (3.00, 6.02)	3.39 (2.48, 4.68)	< 0.001
Cr (µmol/L)	83.11 ± 61.82	87.23 ± 54.99	78.57 ± 68.30	0.016
eGFR	103.69 ± 39.05	98.36 ± 32.18	109.56 ± 44.73	< 0.001
FBG (mmol/L)	4.91 ± 2.88	4.81 ± 3.56	4.15 ± 1.83	< 0.001
Na (mmol/L)	136.13 ± 5.87	135.55 ± 5.24	136.77 ± 6.43	< 0.001
WBC (10^9^/L)	7.09 (5.38, 9.39)	6.72 (4.94, 9.02)	7.31 (5.93, 9.90)	< 0.001
NEU (%)	0.72 ± 0.46	0.71 ± 0.38	0.73 ± 0.54	0.467
PLT (10^9^/L)	117.77 ± 74.32	96.61 ± 54.73	140.97 ± 85.29	< 0.001
AFP (ng/mL)	34.85 (10.07, 96.85)	29.90 (10.21, 96.90)	35.50 (9.44, 102.90)	< 0.001
lgHBV-DNA	5.07 (3.51, 6.53)	4.52 (3.04, 6.12)	5.53 (4.20, 7.04)	< 0.001
CTP score	10.89 ± 1.63	11.31 ± 1.48	10.42 ± 1.67	< 0.001
CTP score < 7 (%)	0 (0)	0 (0)	0 (0)	1
7 ≤ CTP score < 9 (%)	253 (21.5)	83 (13.5)	170 (30.3)	< 0.001
CTP score ≥ 10 (%)	924 (78.5)	533 (86.5)	391 (69.7)	< 0.001
MELD score	25.76 ± 6.67	26.48 ± 6.69	24.97 ± 6.56	< 0.001
MELD-Na score	23.95 ± 12.41	25.55 ± 11.94	22.18 ± 12.69	< 0.001

*TBIL* total bilirubin, *ALB* albumin, *AST* aspartate aminotransferase, *ALT* alanine aminotransferase, *CHE* cholinesterase, *PT* prothrombin time, *AFP* alpha-fetoprotein, *WBC* white blood cells, *INR* international normalised ratio, *Fib* fibrinogen, *PLT* platelets, *Cr* creatinine, *BUN* blood urea nitrogen, *eGFR* estimated glomerular filtration rate, *FBG* fasting blood glucose, *CTP* Child–Turcotte–Pugh, *MELD* model for end-stage liver disease, *95% CI* 95% confidence interval, *OR* odds ratio

### Aetiology of HBV-ACLF

**Fig. 2:**
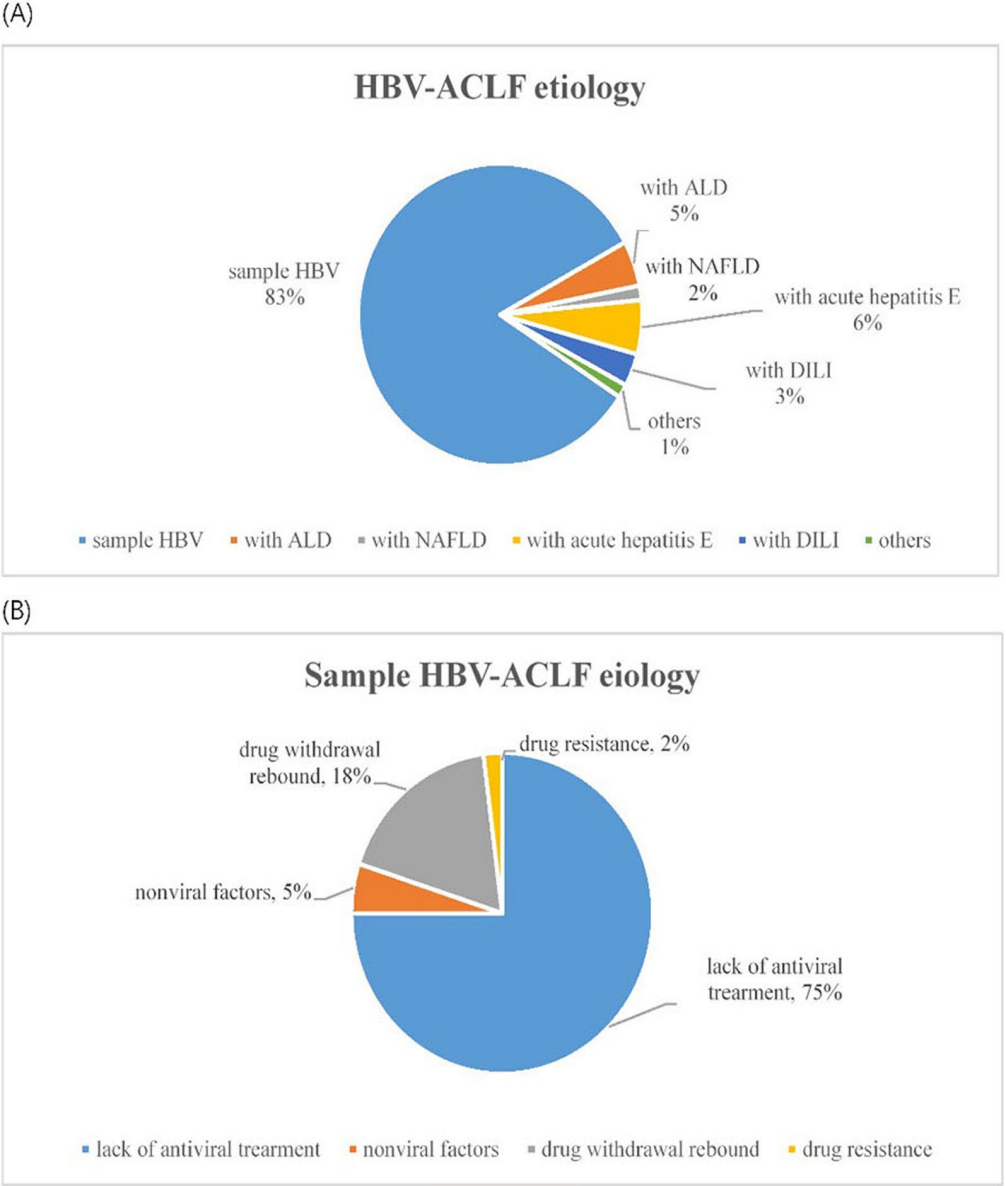
**A** HBV-ACLF aetiology. **B** Simple HBV-ACLF aetiology. HBV-ACLF, hepatitis B virus related acute-on-chronic liver failure; Alcoholic liver disease, ALD; drug-induced liver injury, DILI; non-alcoholic fatty liver disease, NAFLD

Among the 1177 patients in this study, HBV-ACLF was associated with HBV alone in 973 (83%) patients and with two or more aetiological factors in 204 (17%) patients. The top three causes were as follows: concomitant acute hepatitis E (68 cases, 6%), alcoholic liver disease (ALD) (57 cases, 5%), and drug-induced liver injury (DILI) (41 cases, 3%) (Fig. 2A). As revealed by further analysis of the precipitating events in patients with simple HBV-ACLF, the leading cause was lack of antiviral treatment (734 cases; 75%), followed by self-withdrawal of NA (171 cases; 18%), antiviral drug resistance (21 cases; 2%), and non-viral factors (47 cases; 5%) (Fig. 2B).

### Ninety-day transplantation-free mortality of HBV-ACLF patients

**Fig. 3:**
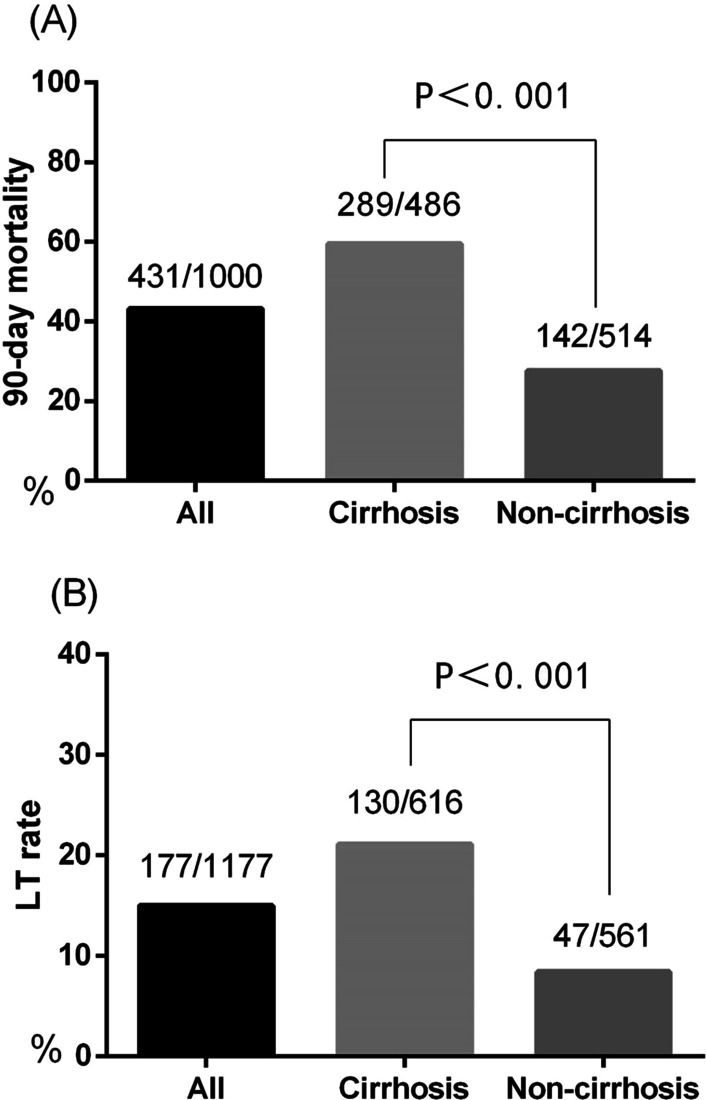
**A** Ninety-day transplantation-free mortality in HBV-ACLF patients. **B** Liver transplantation rate in HBV-ACLF patients. Liver transplantation, LT

Of the 1177 HBV-ACLF patients in the study, 130 (21.1%) patients with cirrhosis and 47 (8.4%) patients without cirrhosis received LT within 90 days. A total of 431 (43.1%) patients died within 90 days, including 289 patients with cirrhosis and 142 patients without cirrhosis. The 90-day LT-free mortality in the cirrhosis group was significantly higher than that in the non-cirrhosis group (59.5% vs. 27.6%, *P* < 0.001) (Fig. 3).

### Occurrence of clinical adverse events

The cirrhosis group had a significantly higher incidence of new decompensated liver cirrhosis, ACLF recurrence and HCC than the non-cirrhosis group (15.7% vs. 3.0%, 3.6% vs. 0.8%, 3.6% vs. 0.5%, *P* < 0.05) (Table 2).

**Table 2:** Occurrence of clinical adverse events

	Total (n = 569)	Cirrhosis (n = 197)	Non-cirrhosis (n = 372)	*P* value
Decompensated cirrhosis	42 (7.4)	31 (15.7)	11 (3.0)	< 0.001
ACLF	10 (1.8)	7 (3.6)	3 (0.8)	0.018
HCC	9 (1.6)	7 (3.6)	2 (0.5)	0.006

Acute-on-chronic liver failure, ACLF; hepatocellular carcinoma, HCC

### Cumulative survival rate of HBV-ACLF patients

**Fig. 4:**
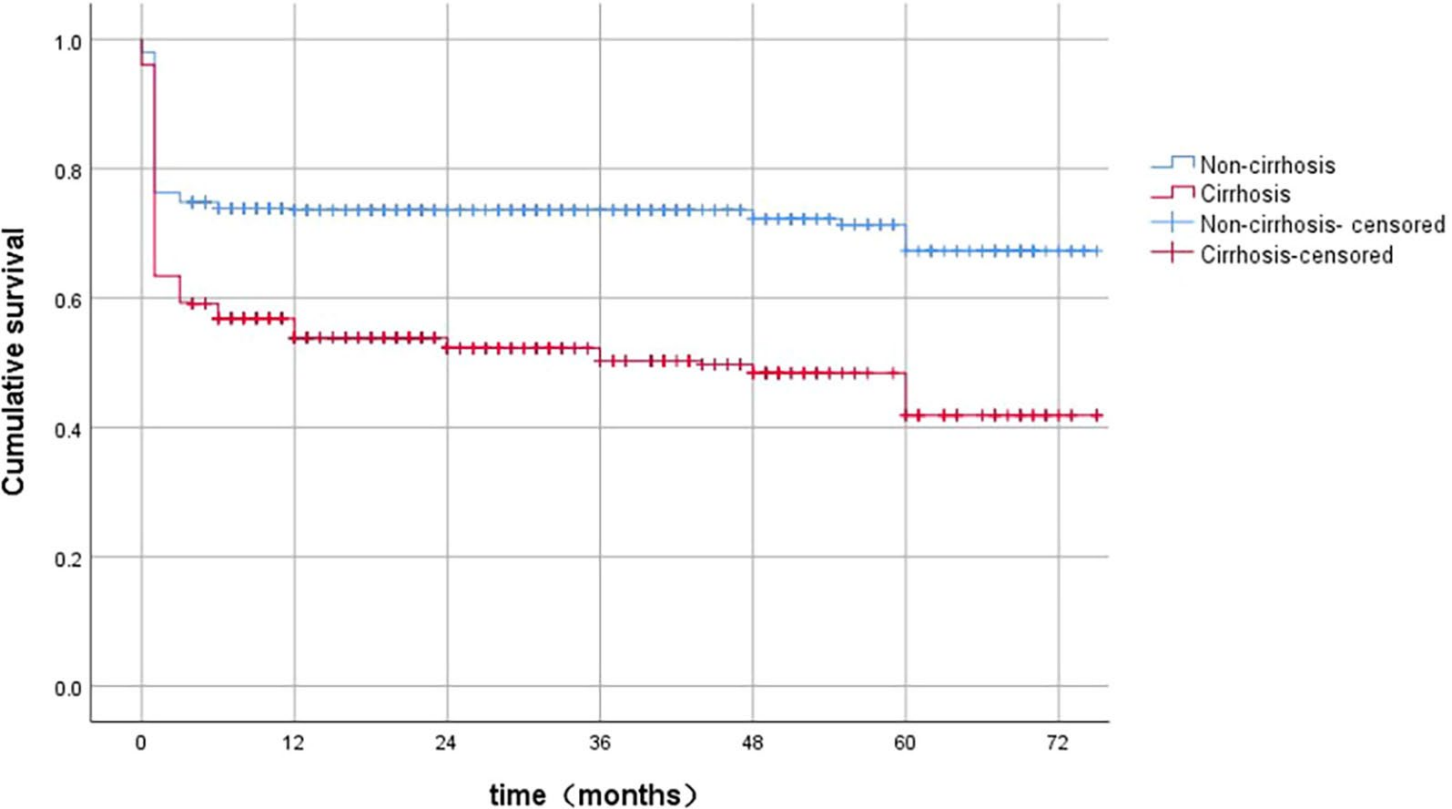
The cumulative survival rates in HBV-ACLF patients

The cumulative survival rates of the overall patients at 12, 36, and 60 months were 63%, 61%, and 50%, respectively. The corresponding cumulative survival rates at 12, 36, and 60 months in the cirrhosis group were significantly lower than those in the non-cirrhosis group (55%, 45%, and 36% vs. 72%, 69%, and 62%; log-rank test, *P* < 0.001) (Fig. 4).

### Risk factors for 90-day mortality in HBV-ACLF patients

We assessed the correlation between clinical parameters and 90-day mortality in HBV-ACLF patients (Table 3). Univariate logistic regression analysis revealed significant between-group differences in age, liver cirrhosis at admission, HE, NA withdrawal, AST level, CHE level, TBIL level, PT level, Cr level, PLT count, FBG level, MELD score, and ALSS therapy (*P* < 0.05). These variables were then included in multivariate regression. In the multivariate logistic regression analysis, age, HE, liver cirrhosis at admission, NA withdrawal, TBIL level, and PT level were identified as independent predictors of 90-day mortality in HBV-ACLF patients (*P* < 0.05).

**Table 3:** Risk factors for 90-day mortality in HBV-ACLF patients

	Univariate analysis	*P* value	Multivariate analysis	*P* value
	COR (95% CI)		AOR (95% CI)	
Age	1.060 (1.047, 1.074)	< 0.001	1.049 (1.035, 1.063)	< 0.001
Cirrhosis	2.190 (1.694, 2.839)	< 0.001	1.906 (1.448, 2.509)	< 0.001
HE	5.061 (3.718, 6.934)	< 0.001	3.545 (2.520, 4.986)	< 0.001
NA withdrawal	1.665 (1.165, 2.374)	0.005	1.845 (1.267, 2.686)	0.002
HBV + ALD	0.484 (0.245, 0.956)	0.037		
AST	1.000 (1.000, 1.000)	0.002		
CHE	1.076 (1.059, 1.094)	< 0.001		
TBIL	1.003 (1.003, 1.004)	0.001	1.003 (1.002, 1.004)	< 0.001
PT	1.486 (1.317, 1.684)	< 0.001	1.054 (1.037, 1.072)	< 0.001
Cr	0.978 (0.973, 0.982)	< 0.001		
FBG	0.924 (0.898, 0.950)	< 0.001		
PLT	0.998 (0.997, 0.998)	< 0.001		
lgHBV DNA	1.186 (1.155, 1.220)	< 0.001		
MELD score	1.155 (1.127, 1.183)	< 0.001		
ALSS	0.868 (0.766, 0.977)	0.022		

*HE* hepatic encephalopathy, *NA* nucleos(t)ide analog, *ALD* alcoholic liver disease, *AST* aspartate aminotransferase, *CHE* cholinesterase, *TBIL* total bilirubin, *PT* prothrombin time, *Cr* creatinine, *PLT* platelets, *FBG* fasting blood glucose, *MELD* model for end-stage liver disease, *ALSS* artificial liver support system, *AOR* adjusted odds ratio, *COR* crude odds ratio

### Risk factors for long-term prognosis of HBV-ACLF patients

The baseline clinical data of patients were analysed in a logistic regression model. The results showed that age, liver cirrhosis at admission, and PLT count were independent risk factors for disease progression, consistent with the risk factors for 90-day mortality. Remarkably, liver cirrhosis at admission (3.675, 95% CI: 2.408–6.594) was a vital risk factor for long-term outcomes in the HBV-ACLF patients. PT level, TBIL level, and MELD score showed no significant correlation with long-term prognosis of HBV-ACLF patients (Table 4).

**Table 4:** Risk factors for long-term prognosis in HBV-ACLF patients

	Univariate analysis	*P* value	Multivariate analysis	*P* value
	COR (95% CI)		AOR (95% CI)	
Age	1.055 (1.034, 1.077)	< 0.001	1.043 (1.018, 1.068)	0.001
Cirrhosis	3.973 (2.553, 6.352)	< 0.001	3.675 (2.408, 6.594)	< 0.001
AST	0.999 (0.999, 1.000)	0.006		
CHE	1.000 (1.000, 1.000)	< 0.001		
TBIL	1.001 (1.000, 1.002)	0.210		
PT	1.004 (0.985, 1.019)	0.603		
Cr	1.002 (0.999, 1.005)	0.197		
PLT	0.992 (0.988, 0.996)	< 0.001	0.994 (0.989, 0.999)	0.020
lgHBV DNA	0.865 (0.776, 0.963)	0.009		
MELD score	1.075 (1.031, 1.120)	0.001		
ALSS	0.542 (0.283, 0.963)	0.049		

*AST* aspartate aminotransferase, *CHE* cholinesterase, *TBIL* total bilirubin, *PT* prothrombin time, *Cr* creatinine, *PLT* platelets, *MELD* model for end-stage liver disease, *ALSS* artificial liver support system, *AOR* adjusted odds ratio, *COR* crude odds ratio

## Discussion

ACLF is an acute hepatic injury syndrome occurring in the backdrop of chronic hepatitis or cirrhosis. This study focused on the 5-year survival rate and risk factors for adverse outcomes in patients with HBV-ACLF in southern China.

HBV reactivation is the primary cause of acute injury in HBV-ACLF patients in Asia; conversely, infection is the leading cause in western countries [13]. Compared with the previous studies [5], the proportion of patients combined with ALD in this study was significantly lower (5% vs. 15.1%), and the incidence of hepatitis E superinfection was higher (5% vs. 2.1%). Furthermore, our results suggested that HBV-ACLF combined with other liver diseases had no significant effect on the 90-day transplantation-free mortality. Significantly, the proportion of patients receiving antiviral treatment for HBV in our cohort was extremely low (20.3%), which is higher than the national figures for China (10.8%) [14]. According to the guidelines [8], antiviral therapy is recommended for cirrhosis patients with HBV. However, only 27.9% of patients with cirrhosis received antiviral therapy prior to admission in our cohort. There are two main causes. On the one hand, many patients do not pay attention to their illness before the onset of ACLF, and they lack regular follow-up and antiviral treatment. On the other hand, with the rapid development of medical technology, the level of medical treatment and clinicians in southern China is still unbalanced. Therefore, some patients with cirrhosis were misdiagnosed and missed, which eventually resulted in not receiving antiviral treatment on time. Moreover, compared with the non-cirrhosis group, the proportion of patients who self-discontinued antiviral therapy was higher in the cirrhosis group. Indeed, these patients have poor medication compliance and lack regular follow-up. Given the status of antiviral therapy for HBV in China, it is crucial to implement a strategy for screening and disseminating antiviral drugs across the country, so as to help reduce the incidence of HBV-ACLF.

Worldwide, there have been many clinical studies on patients with ACLF. Because of the different definitions of ACLF used in eastern and western countries, there are no standardised diagnostic criteria and prognostic models for these patients. Compared with a European study, which was based on the European Association for the Study of the Liver Chronic Liver Failure Consortium (EASL CLIF-C) criteria, the 90-day LT-free mortality of ACLF patients was slightly higher in our study (59.5% vs. 51.2%) [15]. Another study from Korea reported a 90-day mortality of 42.4% in ACLF patients who satisfied the APASL definition [16]. However, the results as mentioned above are inconsistent with the Chinese Group on the Study of Severe Hepatitis B (COSSH) study, which was conducted between 2013 and 2016 [17]. The patients with ACLF based on the COSSH criteria showed a higher 90-day mortality than the patients in our study (69.7% vs. 59.5%). Moreover, we found that the 5-year cumulative mortality in the entire sample was quite close to the 90-day transplantation-free mortality (50.0% vs. 43.1%), which indicated that HBV-ACLF patients who survived more than 90 days had a favourable prognosis in the late stage. Recent data from Korea showed that the cumulative 1-year mortality rate for patients with ACLF is 32%, which is similar to that in our study [18]. Compared with another cohort study in China [6], the long-term survival in our study was significantly lower (62% vs. 97.2%), which may be attributed to the exclusion of patients who died within 3 months from the previous study. At present, there are few reports of a 5-year cumulative survival rate in patients with HBV-ACLF in China and abroad. Collectively, despite the development of antiviral drugs and ALSS therapy in recent years, the outcomes of ACLF have not improved significantly compared with those reported by previous studies.

Most notably, our results showed that liver cirrhosis at admission was an independent risk factor for both short-term and long-term outcomes. Several studies have found a correlation between cirrhosis and poor prognosis in patients with HBV-ACLF [19]. Of note, withdrawal of NA is an important acute injury event [20], and its incidence has been increasing in recent years. Consistent with our study, Shi et al. [21] found that HBV-ACLF patients who self-discontinued NA showed a poor short-term prognosis. However, there is no prognostic model for HBV-ACLF patients, including the intrahepatic precipitating factor. Therefore, clinicians should ascribe great importance to the influence of intrahepatic causes on the prognosis of HBV-ACLF.

This was a sizeable single-centre retrospective study with certain limitations. First, due to the different aetiologies and disease characteristics of ACLF in eastern and western countries, ACLF patients in our cohort were diagnosed based on the APASL diagnostic criteria but not based on the EASL-CLIF standards, which may limit the generalisability of our results. Second, lactic acid level was not routinely monitored in our centre in the past, which resulted in the lack of relevant data. Thus, it is difficult to compare the AARC and CLIF-SOFA scores with the MELD score in evaluating the prognosis of patients. We intend to increase the detection of lactic acid in subsequent studies. Third, the rate of loss to follow-up was relatively high; thus, the results may have been influenced by recall bias. These limitations of the retrospective study need to be resolved in a multi-centre prospective study in the future.

In summary, the proportion of HBV-ACLF patients receiving antiviral treatment is very low in southern China. Cirrhosis at admission has a significant effect on short-term and long-term prognosis. No significant improvement in the short-term prognosis of HBV-ACLF patients was observed compared with previous studies. More comprehensive access to antiviral treatment and long-term surveillance of HBV patients are key imperatives to reduce the incidence of ACLF and improve the prognosis.

## Data Availability

The data used to support the findings of this study are available from the corresponding author upon request.

## Abbreviations

HBV: Hepatitis B virus
CHB: Chronic hepatitis B
ACLF: Acute-on-chronic liver failure
ALSS: Artificial liver support system
HCC: Hepatocellular carcinoma
LT: Liver transplantation
HE: Hepatic encephalopathy
ALD: Alcoholic liver disease
DILI: Drug-induced liver injury
NAFLD: Nonalcoholic fatty liver disease
NA: Nucleos(t)ide analog
TBIL: Total bilirubin
ALB: Albumin
AST: Aspartate aminotransferase
ALT: Alanine aminotransferase
CHE: Cholinesterase
PT: Prothrombin time
AFP: Alpha-fetoprotein
WBC: White blood cells
INR: International normalised ratio
Fib: Fibrinogen
PLT: Platelets
Cr: Creatinine
BUN: Blood urea nitrogen
eGFR: Estimated glomerular filtration rate
FBG: Fasting blood glucose
CTP: Child–Turcotte–Pugh
MELD: Model for end-stage liver disease
APASL: Asian Pacific Association for the Study of Liver
COSSH: The Chinese Group on the Study of Severe Hepatitis B
EASL CLIF-C: The European Association for the Study of the Liver Chronic Liver Failure Consortium
CI: Confidence interval
OR: Odds ratio
AOR: Adjusted odds ratio
COR: Crude odds ratio
